# Publisher Correction: Antagonism between brain regions relevant for cognitive control and emotional memory facilitates the generation of humorous ideas

**DOI:** 10.1038/s41598-021-97696-4

**Published:** 2021-09-06

**Authors:** Florian Bitsch, Philipp Berger, Andreas Fink, Arne Nagels, Benjamin Straube, Irina Falkenberg

**Affiliations:** 1 Department of Psychiatry and Psychotherapy, Philipps-University Marburg, Rudolf-Bultmann-Straße 8, 35039 Marburg, Germany; 2 Center for Mind, Brain and Behavior - CMBB, Hans-Meerwein-Straße 6, 35032 Marburg, Germany; 3 Department of Neuropsychology, Max Planck Institute for Human Cognitive and Brain Sciences, Stephanstraße 1a, 04103 Leipzig, Germany; 4 Institute of Psychology, University of Graz, BioTechMed, Universitätsplatz 2, 8010 Graz, Austria; 5 Department of English and Linguistics, Johannes Gutenberg-University Mainz, Jakob-Welder-Weg 18, 55128 Mainz, Germany

Correction to: *Scientific Reports* 10.1038/s41598-021-89843-8, published online 21 May 2021

In the original version of the Article, Fig. 4 was a duplication of Fig. 5.

In addition, the Introduction contained a duplication error, where

“To examine whether inhibitory mechanisms modulate the mining of humorous ideas, we assessed each participant’s cognitive control capacities with a color naming stroop task outside the MRI and examined their potential effect on the idea generation process. We hypothesize that brain regions relevant for processing humor, such as the prefrontal cortex, the amygdala and the hippocampus, are implicated in the generation of funny (vs. typical) ideas.”

now reads:

“To examine whether inhibitory mechanisms modulate the mining of humorous ideas, we assessed each participant’s cognitive control capacities with a color naming stroop task outside the MRI and examined their potential effect on the idea generation process.”

“We hypothesize that brain regions relevant for processing humor, such as the prefrontal cortex (particularly the SFG), the amygdala and the hippocampus, are implicated in the generation of funny (vs. typical) ideas. This hypothesis will be tested by contrasting the functional activity during the generation of funny > typical ideas. Furthermore, we expected that these brain regions and particularly prefrontal brain regions are modulated by inhibitory mechanisms which will facilitate the generation of humorous ideas. This hypothesis will be tested by investigating the effects of inhibitory abilities, as assessed by a stroop-task, during the generation of funny ideas. Based on studies showing the high relevance of the superior/medial frontal gyrus^29^ and the amygdala, we assumed that the functional connectivity between both regions will facilitate the flourishing of humorous ideas. Explorative analysis will examine whether there is an association between the production of funny ideas and personality differences in the regulation of emotional responses with the amygdala and superior/medial frontal gyrus.”

now reads:

“We hypothesize that brain regions relevant for processing humor, such as the prefrontal cortex (particularly the SFG), the amygdala and the hippocampus, are implicated in the generation of funny (vs. typical) ideas.”

The original Article has been corrected.

