# Supplementary material for: Antagonism between brain regions relevant for cognitive control and emotional memory facilitates the generation of humorous ideas
Source: Sci Rep. 2021 May 21;11:10685. doi: 10.1038/s41598-021-89843-8 (PMC8140114; doi:10.1038/s41598-021-89843-8)
Supplement: Supplementary file 1 — Supplementary Information. [file 41598_2021_89843_MOESM1_ESM.docx]

**Supplementary Material**

**Antagonism Between Brain Regions Relevant for Cognitive Control and Emotional Memory Facilitates the Generation of Humorous Ideas**

Florian Bitsch^1,2^*, Philipp Berger^1,3^, Andreas Fink^4^, Arne Nagels^1,5^, Benjamin Straube^1,2^, &
 Irina Falkenberg^1,2^

1 Department of Psychiatry and Psychotherapy, Philipps-University Marburg, Rudolf-Bultmann Str. 8,
 35039 Marburg, Germany

2 Center for Mind, Brain and Behavior - CMBB, Hans-Meerwein-Straße 6, 35032, Marburg, Germany

3 Department of Neuropsychology, Max Planck Institute for Human Cognitive and Brain Sciences,
 Stephanstraße 1a, 04103 Leipzig, Germany

4 Institute of Psychology, University of Graz, BioTechMed, Universitätsplatz 2, 8010, Graz, Austria

5 Department of English and Linguistics, Johannes Gutenberg-University Mainz, Jakob-Welder-Weg
 18, 55128 Mainz, Germany

*****Correspondence to:

Florian Bitsch

Philipps-University Marburg

Department of Psychiatry and Psychotherapy

Rudolf-Bultmann-Straße 8, 35039 Marburg, Germany

Tel: +49-6421-58-63604

Email: bitsch@med.uni-marburg.de

**Functional Activity Analysis**

**Additional Figures for the Functional Activity Analysis: FUN>TYP**

1. B)

C) D)

Functional Activity Analysis: The functional activity analysis of the generating FUN>TYP ideas contrast. Parameter estimates (mean beta values per condition) of A) the Calcarine Gyrus, B) the Postcentral Gyrus, C) the Inferior Parietal Lobule and the D) Cerebellum. The error bars represent SEM.

**Additional Information for the Correlation Analysis with the Robust Correlation Toolbox**

*The left amygdala activity and its connectivity with the right superior and right medial frontal gyrus (r=-.57, p<.05; 1 outlier) indicates a stronger functional activity increase of the left amygdala by a higher negative frontal coupling.*

*Correlations of extracted beta estimates showed a significant association between the left amygdala activity with the number of generated ideas in the funny condition (r=0.50, p<.05; 3 outliers).*

*Furthermore, the quantity of participants’ produced ideas during the funny condition correlated negatively with functional connectivity measures of the amygdala-superior/medial frontal gyrus (r=-0.44, p<.05, 1 outlier),*

*A further analysis showed that a suppressive emotion-regulation tendency (ERQ) (r=-0.49, p<.05; 2 outliers) is associated with reduced left amygdala activity during the production of funny ideas.*
